# Attitudes of doctors of medicine and dental medicine towards contacting patients via social networks: Marko Marelic

**DOI:** 10.1093/eurpub/ckac131.170

**Published:** 2022-10-25

**Authors:** D Relic, M Marelic, L Machala Poplasen, J Viskic, K Sedak, M Majer, T Vukusic Rukavina

**Affiliations:** 1 Andrija Stampar School of Public Health, School of Medicine University of Zagreb, Zagreb, Croatia; 2 Department of Fixed Prosthodontics, School of Dental Medicine University of Zagreb, Zagreb, Croatia; 3 Department of Communication Sciences, Catholic University of Croatia, Zagreb, Croatia

## Abstract

**Background:**

As the use of social networking sites (SNSs) has greatly increased among health professionals, it is necessary to investigate their use of SNSs. The aim of this study was to identify the patterns of SNS use for contact with patients between medical doctors (MD) and doctors of dental medicine (DMD) in Croatia.

**Methods:**

In collaboration with the Croatian Medical Chamber and the Croatian Chamber of Dental Medicine, a quantitative cross-sectional study was conducted on the use of SNSs and the attitudes of MDs and DMDs towards e-professionalism. Data were collected using online questionnaires. The data were analyzed using descriptive statistics.

**Results:**

A total of 753 responses were processed, 507 (67,3%) MDs and 246 (32,7%) DMDs. DMDs were significantly more likely to visit patients or their family members’ profiles on SNSs (60,6% vs 33,3%, P < 0.001). The main reason for visiting a patient’s or family member’s profile is social communication, which is significantly more common among DMDs (46,3% vs 29,0%, P < 0.002). DMDs are significantly more likely to send friend requests from private SNS profiles to patients or their family members (15,0% DMDs vs 3,4% MDs, P < 0.001). Patients are much more likely to send friendship and connection requests on SNS to DMDs (91,1% DMDs vs 62,7% MDs, P < 0.001) who are also significantly more likely to accept these requests (76,8% DMDs vs 41,5% MDs, P < 0.001). MDs and DMDs would find the presence of e-professionalism guidelines useful (72,2% MDS vs 78,0% DMDs, P = 0.103).

**Conclusions:**

The results show that DMDs are more open to communicating with patients via SNSs. Results showed a difference between MDs and DMDs in both directions - in sending requests to patients or their family members and in positive responses to their requests for connection. The findings point to the need to develop guidelines for health professionals regarding e-professionalism.

**Key messages:**

• There are differences between MDs and DMDs toward communicating with patients using SNSs, DMDs being more open to the communication with patients.

• There is a need to develop guidelines for health professionals on e-professionalism with emphasis regarding how to professionally communicate with patients on SNSs.

